# Single‐molecule electrical protein fingerprinting in solid‐state nanopores

**DOI:** 10.1002/ctm2.70589

**Published:** 2026-01-07

**Authors:** Neeraj Soni, Amit Meller

**Affiliations:** ^1^ Faculty of Biomedical Engineering Russell Berrie Nanotechnology Institute, Technion ‐ IIT Haifa Israel

1

Proteins play a central role in virtually all biological processes and serve as critical indicators of health and disease. Despite their importance, protein‐based diagnostics remain far less developed than nucleic acid‐based approaches. A major limitation is the insufficient sensitivity of current proteomic technologies, which typically require either enzymatic digestion of proteins into peptides or their immobilisation on surfaces for affinity‐based detection.^1^ These strategies inherently discard information encoded in full‐length proteins and rely heavily on the availability and performance of high‐quality antibodies.

Soni et al.^2^ report a fundamentally different approach for antibody‐free, full‐length protein fingerprinting based on solid‐state nanopores. A nanopore is a nanometre‐scale aperture in a thin insulating membrane that separates two electrolyte reservoirs. When a biopolymer translocates through the pore under an applied electric field, it transiently modulates the ionic current, producing a signal that reflects the molecule's physical and chemical properties.^3^ This principle underpins nanopore‐based DNA sequencing, in which variations in ionic current are used to infer the nucleotide sequence of individual DNA molecules. The success of nanopore DNA sequencing is attributable to several intrinsic features of DNA: a uniformly charged backbone, the closely related chemical structures of its four nucleotides and the availability of enzymes that regulate DNA motion through the pore. Extending this framework to proteins, however, is substantially more challenging because of the immense diversity in protein size, charge distribution and three‐dimensional structure. Additionally, unlike DNA, proteins cannot be amplified, imposing stringent requirements on the sensor's limit of detection (LoD).

To overcome key barriers such as single‐file translocation and limited temporal resolution,^4^ Soni et al.^2^ introduce a chemo‐selective labelling strategy (Figure 1). Proteins are denatured and site‐specifically conjugated at cysteine residues with short single‐stranded DNA oligonucleotides (5‐ or 10‐mers) using click chemistry. Remarkably, proteins labelled with a 5‐mer oligonucleotide exhibit a distinctive stick–slip translocation mechanism, characterised by prolonged transient binding within the nanopore at the oligonucleotide–cysteine junction. This behaviour is absent in unconjugated proteins and results in an approximately 20‐fold slowing of translocation dynamics. Molecular dynamics simulations corroborate the mechanistic origin of these interactions. This controlled slowdown enables the generation of time‐resolved ionic current pulses, which serve as proxies for the positions of cysteine residues along the protein backbone. These pulse patterns constitute protein‐specific fingerprints. Using only a few tens of translocation events, dynamic time‐warping analysis produces consensus signal maps, successfully implemented across six distinct proteins.

**FIGURE 1:**
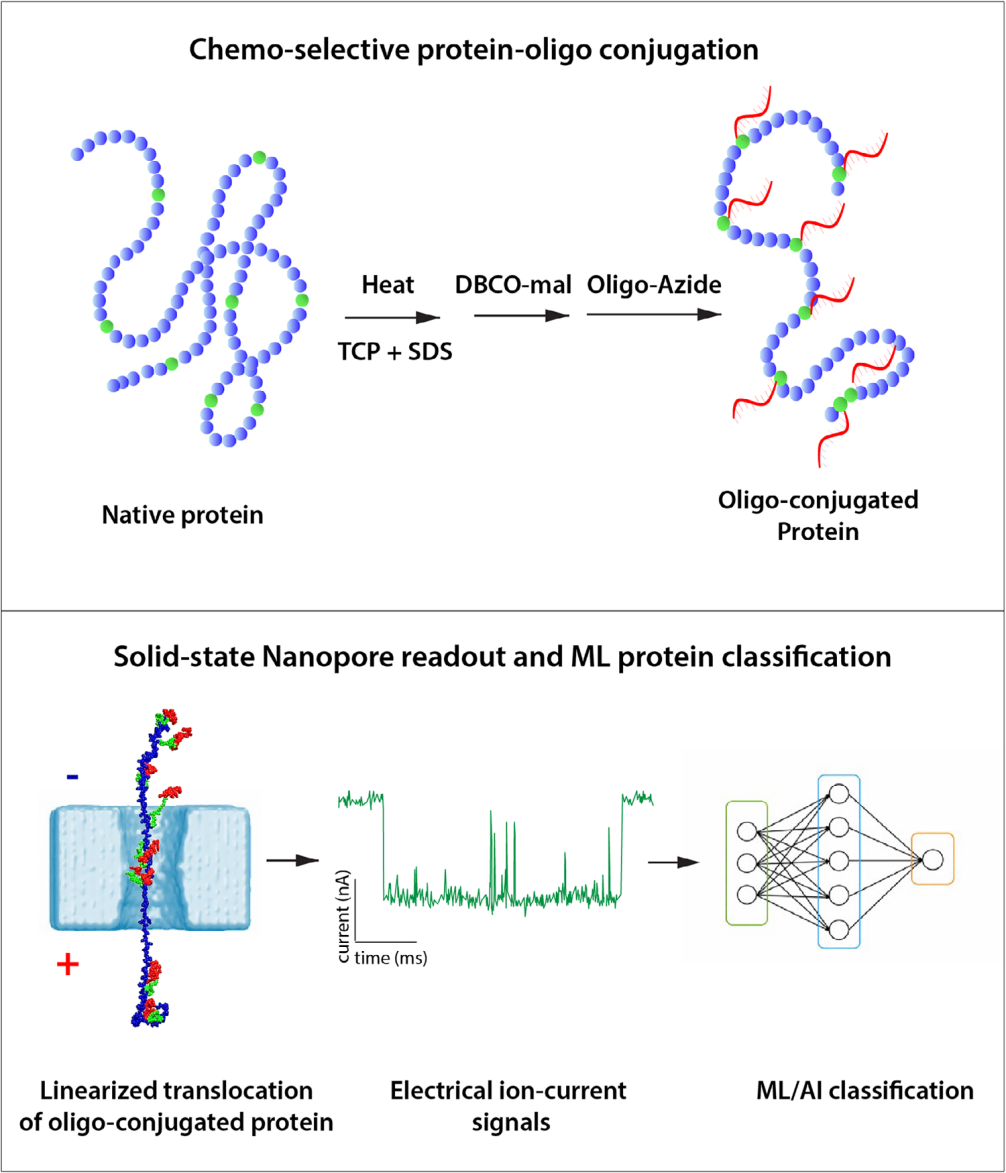
Top: Schematic representation of protein denaturation achieved through reduction with TCEP, heating and SDS treatment, followed by the chemo‐selective labelling of cysteine residues using DBCO–maleimide to facilitate click chemistry for oligo conjugation. Bottom: Linearised protein translocation from (−) to (+) biased sides, with protein (blue), linker (green) and oligo (magenta). A representative ionic current blockade trace is shown, and the extracted signal features are subsequently processed by a machine‐learning‐based classification framework for protein identification.

The clinical relevance of this approach is further demonstrated using two isoforms of vascular endothelial growth factor. Conventional nanopore metrics ‘blockade current’ and ‘dwell time’ show complete overlap between the isoforms, rendering discrimination infeasible. In contrast, the number and temporal arrangement of conductive pulses provide a robust feature for isoform‐level differentiation, even when just a few copies of the proteins are sampled by the nanopore. Moreover, machine‐learning‐assisted classification enables accurate identification of target proteins even within complex mixtures.

Beyond fingerprinting, oligonucleotide conjugation substantially enhances protein delivery to the nanopore due to the added negative charge. This is particularly significant for clinical samples, where many protein biomarkers are present at extremely low endogenous concentrations and, unlike nucleic acids, cannot be amplified. Soni et al.^2^ demonstrate a >10‐fold increase in capture rate following oligo conjugation, which directly translate to an order of magnitude boost in the nanopore LoD.

Emerging biological nanopore approaches for full‐length protein analysis, whether enzyme‐guided or enzyme‐free, have achieved improved resolution, but often at the expense of delivery efficiency.^5^ By contrast, the present work attains high sensitivity while simultaneously improving the nanopore's LoD, all without relying on enzymatic motors. Together, these results establish a scalable, antibody‐free nanopore platform capable of identifying full‐length proteins at ultralow concentrations. This strategy marks a significant step toward clinically translatable proteomic diagnostics, providing a pathway to highly sensitive, label‐minimal and information‐rich protein detection directly from complex biological samples.

## CONFLICT OF INTEREST STATEMENT

The authors declare no conflicts of interest.

## References

[1] Alfaro JA , Bohländer P , Dai M , et al. The emerging landscape of single‐molecule protein sequencing technologies. Nat Methods. 2021;18:604. 34099939 10.1038/s41592-021-01143-1 PMC8223677

[2] Soni N , Rosenstock Z , Verma NC , et al. Full‐length protein classification via cysteine fingerprinting in solid‐state nanopores Nat Nanotechnol. 2025;20:1482. 40993352 10.1038/s41565-025-02016-w PMC13272770

[3] Kasianowicz JJ , Brandin E , Branton D , Deamer DW , in Proc. Natl. Acad. Sci. U. S. A., National Academy of Sciences, 1996, pp. 13770‐13773. 10.1073/pnas.93.24.13770 PMC19421 8943010

[4] Soni N , Freundlich N , Ohayon S , Huttner D , Meller A , Single‐File Translocation Dynamics of SDS‐Denatured, Whole Proteins through Sub‐5 nm Solid‐State Nanopores ACS Nano. 2022;16:11405. 35785960 10.1021/acsnano.2c05391 PMC7613183

[5] Ritmejeris J , Chen X , Dekker C . Nat Rev Bioeng. 2024;3:303. 2024 34.

